# Non-uniform Stress-free Strains in a Spherically Symmetrical Nano-sized Particle and Its Applications to Lithium-ion Batteries

**DOI:** 10.1038/s41598-018-23320-7

**Published:** 2018-03-21

**Authors:** Qingping Meng, Lijun Wu, David O. Welch, Ming Tang, Yimei Zhu

**Affiliations:** 1 Brookhaven National Laboratory, Upton, New York 11973 USA; 2 Department of Materials Science & Nanoengineering, Rice University, Houston, USA

## Abstract

The stress-free strain originated from local chemical composition and phase transformation can significantly alter the microstructures of materials; and then affect their properties. In this paper, we developed an analytical method to calculate stress-strain field due to the non-uniform stress-free strain in a spherically symmetrical particle. Applying the method to a lithium ion (Li-ion) battery electrode, the evolution of Li-ion concentration and strain field during the lithiation process is studied. Our studies reveal that the maximum strain in the electrode generally occurs on surface of sample, and is mainly dependent on the difference of Li-ion concentration of surface and of center in sample. Decreasing the difference of Li-ion concentration can efficiently decrease the maximum strain so that cracks of electrodes can been prevented. Our analytical results provide a useful guidance for practical applications of energy storage materials.

## Introduction

The properties of solids depend on their microstructure, chemical composition, lattice structure, and even size^1^. The knowledge of phase transformation^2–4^ tells us that the misfit stress-free strain of a newly formed phase in a matrix can seriously affect the behavior of phase transformation and microstructure of the material, such as the autocatalytic nucleation of martensitic transformation in bulk^3^ and nucleation barrier of phase transformation in nano-sized materials^4^. In Li-ion batteries, the microstructures are more complex because of non-conservation of chemical composition and more occurring phase transformations during insertion and extraction of Li-ions^5,6^. Many experiments and theories have proved that the stress-strain field generated by misfit stress-free strain between the lithiated and delithiated regions can significantly influence the evolution of phase morphology in Li-ion batteries^7–12^. For example, the stress changes the behavior of phase transformation^7,8^ and Li-ion diffusion-rate^9^ and induces fracture of electrode^10–12^.

**Figure 1:**
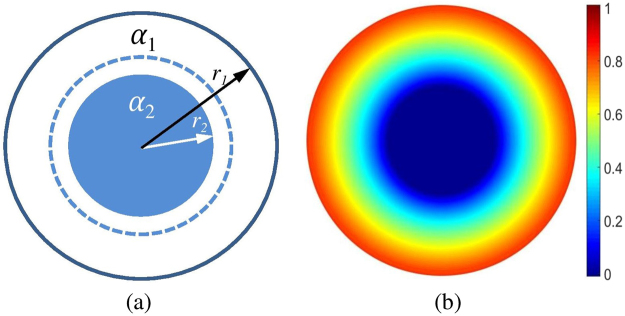
A spherical particle consisting of (**a**) two phases of α1 and α2 phase, and (**b**) gradient distribution of concentration. The dished line in (**a**) is the profile of α1 phase before its change. The color bar in (**b**) shows the change of concentration.

Non-uniform distribution of Li-ion concentration will cause stress-strain field in electrodes of lithium batteries. The stress-strain field is very sensitive to the high-rate of Li-ion exchange^13,14^. The high-rate exchange of Li-ion can generate steep gradients of stresses and strains that ultimately lead to fracture of electrodes and performance degradation of batteries^13,14^. In order to explain the fracture of electrode, Christensen and Newman^10,11^ calculated the stress-strain field in active electrode material of Li-ion cell using a similar model used by Meng *et al*.^4^. They predict that fracture of Li_y_Mn_2_O_4_ depends only upon the ratio of the two phases, LiMn_2_O_4_/Li_2_Mn_2_O_4_, as if they do not relate to the size of electrode. Christensen and Newman’s conclusion is not consistent with the experimental observations^15,16^. Analysis indicates that Christensen and Newman’s calculations have two issues: (1) the model in their calculations only includes two uniform regions (see Fig. 1(a))^4,10,11^. In α_1_ and α_2_ region shown in Fig. 1(a), the chemical composition is uniform. For real Li-ion electrodes, the distribution of Li-ion concentration is non-uniform with a gradient from surface to center of electrode during lithiation and delithiation. Figure 1(b) schematically shows the non-uniform distribution. Consequently, the calculated results based on the model shown in Fig. 1(a) will have its limitation. (2) The evolution of stress-strain field with time was not considered; therefore, the effect of diffusion velocity of Li-ion and size of electrode was not included in Christensen and Newman’s calculations.

A number of theoretical models have been developed to obtain a strain field of non-uniform distribution^17–20^. Johnson^17^ uses his model to study the spinodal decomposition for small, isotropic, radially symmetric spherical and mass conservation system. Using similar to Johnson’s theory, other researchers^18–20^ calculated the stresses and strains in Li-ion electrodes. In addition to these analytical calculations^18–20^, some numerical methods using commercial software package COMSOL^21^ and ABAQUS finite element package^22^ are used to study Li-ion battery. Comparing these analytical calculations^18–20^ with some numerical calculated results^21,22^, some discrepancies are found. For example, the stress field is always a monotonic function of radius in analytical calculations, but it is not in numerical methods. In this study, we develop a new analytical method on the stress-strain field of the non-uniform concentration distribution in a symmetrical spherical particle and mass non-conservation system, as shown in Fig. 1(b). Although this geometrical constraint is not realized in many experimental systems, the results of the analysis provide insight and guidance for actual material systems. In our method, a general solution is obtained, and some assumptions which should be made in the prior analytical and numerical method, such as elastic modulus non-uniformity, are not necessary using our derivations. Further, we shall apply our model to the strain field in battery electrode during lithiation process and discuss these calculated results.

## The mathematical model of non-uniformed strain

### Mathematical model

The traditional method of calculating restricted strain from known stress-free strains is Eshelby’s inclusion theory^23,24^. This theory assumes that an inclusion with uniform stress-free strains was embedded in an infinite homogeneous body. Using Eshelby’s theory for our issue, a two-layered shell-structured spherical particle is first considered, as shown in Fig. 1(a). We assume that the inner part *α*_2_ of the particle will have a stress-free volumetric strain *ε*_2_ relative to the outside part *α*_1_ when Li-ions are inserted; and then the radius of the inner part *α*_2_ will change from the original radius *r*_2_ to

1 $${r}_{2}^{{\alpha }_{2}}=(1+{\varepsilon }_{2}){r}_{2}$$

According to Eshelby’s method^23,24^, for spherical symmetry, we know that the stress field is a function of radius *r* only. Therefore, the equation of displacement equilibrium^25^ is

2 $$\frac{{\partial }^{2}\omega }{\partial {r}^{2}}+\frac{2}{r}\frac{\partial \omega }{\partial r}-\frac{2\omega }{{r}^{2}}=0$$

where *ω* is the displacement. The general solution of Eq. (2) is

3 $$\omega ={C}_{1}r+\frac{{C}_{2}}{{r}^{2}}$$

The values of *C*_1_ and *C*_2_ can be determined from the boundary conditions. In the inner part *α*_2_, *C*_2_ = 0 because the displacement *ω* should remain finite as *r* → 0. The displacements *ω* in the two parts respectively are

4.1 $${\omega }^{{\alpha }_{1}}={C}_{1}^{{\alpha }_{1}}r+\frac{{C}_{2}^{{\alpha }_{1}}}{{r}^{2}}$$

4.2 $${\omega }^{{\alpha }_{2}}={C}^{{\alpha }_{2}}{\varepsilon }_{2}r$$

From Eq. (4.2), the displacement of the free strain in the *α*_2_ resulting from the constraint of *α*_1_ is $({C}^{{\alpha }_{2}}-1){\varepsilon }_{2}$, and, for this state, the stress in *α*_2_^26,27^ is

5 $$P=3{K}^{{\alpha }_{2}}({C}^{{\alpha }_{2}}-1){\varepsilon }_{2}$$

The stress in the *α*_1_ is found to be as follows:

6.1 $${\sigma }_{rr}^{{\alpha }_{1}}=(3{\lambda }^{{\alpha }_{1}}+2{\mu }^{{\alpha }_{1}}){C}_{1}^{{\alpha }_{1}}-\frac{4{\mu }^{{\alpha }_{1}}}{{r}^{3}}{C}_{2}^{{\alpha }_{1}}$$

6.2 $${\sigma }_{\theta \theta }^{{\alpha }_{1}}={\sigma }_{\varphi \varphi }^{{\alpha }_{1}}=(3{\lambda }^{{\alpha }_{1}}+2{\mu }^{{\alpha }_{1}}){C}_{1}^{{\alpha }_{1}}+\frac{2{\mu }^{{\alpha }_{1}}}{{r}^{3}}{C}_{2}^{{\alpha }_{1}}$$

where ${K}^{{\alpha }_{2}}$, ${\lambda }^{{\alpha }_{1}}$, and ${\mu }^{{\alpha }_{1}}$ are the bulk modulus of *α*_2_ and the Lamé constant of *α*_1_, respectively. The coefficients ${C}_{1}^{{\alpha }_{1}}$, ${C}_{2}^{{\alpha }_{1}}$, and ${C}^{{\alpha }_{2}}$ are obtained as follows from the boundary conditions.

The mechanical balance of forces at the interface^28^

7 $${{\boldsymbol{\sigma }}}^{\alpha }\cdot {{\bf{n}}}^{\alpha }+{{\boldsymbol{\sigma }}}^{\beta }\cdot {{\bf{n}}}^{\beta }-\nabla \cdot {\bf{f}}=0$$

where the superscripts *α* and *β* denote, respectively, the two different regions on the opposite sides of an interface, ***σ***^*α*^ and ***σ***^*β*^ are the stress tensors of these two regions, respectively, and **n**^*α*^ and **n**^*β*^ are the exterior normal to the *α* and *β* sides of the interface (**n**^*α*^ = −**n**^*β*^), respectively. **f** is the surface’s stress tensor. In the case of spherical symmetry, the components of **f** are **f**_*θθ*_ = **f**_*rr*_^26^ and

8 $$\nabla \cdot {\bf{f}}=-\frac{2f{{\bf{n}}}^{\beta }}{{r}_{i}}$$

where *r*_*i*_ is the radius of the spherical interface. The subscripts *i* =  1, 2 correspond, respectively, to the surface of the particle and the interface of *α*_1_/*α*_2_. The equations of mechanical balance of the particle surface and the *α*_1_/*α*_2_ interface are obtained from Eq. (7).

9.1 $$(3{\lambda }^{{\alpha }_{1}}+2{\mu }^{{\alpha }_{1}}){C}_{1}^{{\alpha }_{1}}-\frac{4{\mu }^{{\alpha }_{1}}}{{r}_{1}^{3}}{C}_{2}^{{\alpha }_{1}}+\frac{2f}{{r}_{1}}=0$$

9.2 $$(3{\lambda }^{{\alpha }_{1}}+2{\mu }^{{\alpha }_{1}}){C}_{1}^{{\alpha }_{1}}-\frac{4{\mu }^{{\alpha }_{1}}}{{r}_{2}^{3}}{C}_{2}^{{\alpha }_{1}}=3{K}^{{\alpha }_{2}}({C}^{{\alpha }_{2}}-1){\varepsilon }_{2}$$

In Eq. (9.2), we ignore the interfacial stress of the *α*_1_/*α*_2_ interface because we assume the changes of chemical compositions or of phase structure between the two sides of an interface are so small that we will transform discrete variables into continuous ones. The equation of the continuity of the normal displacement of the *α*_1_/*α*_2_ interface can be obtained from Eqs (4–5):

9.3 $${C}_{1}^{{\alpha }_{1}}{r}_{2}+\frac{{C}_{2}^{{\alpha }_{1}}}{{r}_{2}^{2}}={C}^{{\alpha }_{2}}{\varepsilon }_{2}{r}_{2}$$

Solving Eqs (9.1–9.3), ${C}_{1}^{{\alpha }_{1}}$, ${C}_{2}^{{\alpha }_{1}}$, and ${C}^{{\alpha }_{2}}$, respectively, are

10.1 $${C}_{1}^{{\alpha }_{1}}=\frac{{D}_{1}{r}_{1}^{3}{\varphi }_{1}^{(31)}-{D}_{2}{r}_{2}^{3}{\varphi }_{2}^{(31)}}{{r}_{1}^{3}{\phi }_{1}^{(3)}-{r}_{2}^{3}{\phi }_{2}^{(3)}}$$

10.2 $${C}_{2}^{{\alpha }_{1}}={r}_{1}^{3}{r}_{2}^{3}\frac{{D}_{1}{\varphi }_{1}^{(32)}-{D}_{2}{\varphi }_{2}^{(32)}}{{r}_{1}^{3}{\phi }_{1}^{(3)}-{r}_{2}^{3}{\phi }_{2}^{(3)}}$$

103 $${C}^{{\alpha }_{2}}=\frac{{D}_{1}{r}_{1}^{3}{\varphi }_{1}^{(33)}-{D}_{2}({r}_{1}^{3}{\varphi }_{2}^{(33)}+{r}_{2}^{3}{\varphi }_{3}^{(33)})}{{r}_{1}^{3}{\phi }_{1}^{(3)}-{r}_{2}^{3}{\phi }_{2}^{(3)}}$$

where ${D}_{1}=-2f/{r}_{1}$, ${D}_{2}=-3{K}^{{\alpha }_{2}}{\varepsilon }_{2}$, and the expressions for the other parameters, ${\varphi }_{k}^{(ij)}$ and ${\phi }_{i}^{(3)}$ (*i*, *j*, and *k* is 1, 2 or 3), are written in Appendix A. This result was found by others^25,26^ if interfacial stress was not considered. The influence of interfacial stress was discussed by Meng *et al*.^4^. Based on these results in Eqs (10.1–10.3), the displacement, strain, and stress of the shell-structured particle can be determined.

In the above model, the chemical composition or phase structure between the inner and outside regions is a step function, i.e., the change of composition or phase structure is not continuous (shown in Fig. 1(a)). For a continuous change of chemical composition or phase structure shown in Fig. 1(b), such as Li-ion battery, we will develop a new calculated method based on the above model. First, we insert a new core *α*_3_ into the center of the particle, i.e., inside region α_2_. For insertion processing, we use Eshelby’s method^2,23^, described as follows:

1. We cut a concentric sphere *α*_3_ centered on the origin, and take the region *α*_3_ out of the parent particle. During this processing, the stress states of other parts of the original particle are kept unchanged.
2. For this case, there exists a stress $P=3{K}^{{\alpha }_{2}}({C}^{{\alpha }_{2}}-1){\varepsilon }_{2}$ in *α*_3_. If *α*_3_ has a new stress-free transformational strain due to a change of chemical composition or structure, we let the *α*_3_ change in keeping the stress *P* state.
3. Let a surface traction be applied to the cut sphere to restore the shape it had previously. The restoration of the shape of *α*_3_ requires a homogeneous strain *ε*_3_.
4. Let *α*_3_ be reintroduced into the hole left in the parent particle after the *α*_3_ was removed.
5. Weld *α*_3_ back on to the parent particle.
6. Let the system relax by introducing the equilibrium elastic strain into the particle.

After the processing described above, the stress in the *α*_1_ still can be expressed by Eq. (6), but the constants ${C}_{1}^{{\alpha }_{1}}$ and ${C}_{2}^{{\alpha }_{1}}$ must be re-determined by the new boundary conditions. The radial stress in *α*_2_, based on the superposition principle, can be written as follows:

11 $${\sigma }_{rr}^{{\alpha }_{2}}=(3{\lambda }^{{\alpha }_{2}}+2{\mu }^{{\alpha }_{2}}){C}_{1}^{{\alpha }_{2}}-\frac{4{\mu }^{{\alpha }_{2}}}{{r}^{3}}{C}_{2}^{{\alpha }_{2}}+3{K}^{{\alpha }_{2}}({C}^{{\alpha }_{2}}-1){\varepsilon }_{2}$$

where ${C}_{1}^{{\alpha }_{2}}$ and ${C}_{2}^{{\alpha }_{2}}$ are parameters that must be determined from the new boundary conditions, but ${C}^{{\alpha }_{2}}$ has been calculated in Eq.(10.3). The stress in *α*_3_ is

12 $$P=3{K}^{{\alpha }_{3}}({C}^{{\alpha }_{3}}-1)({\varepsilon }_{3}-{\varepsilon }_{2})+3{K}^{{\alpha }_{2}}({C}^{{\alpha }_{2}}-1){\varepsilon }_{2}$$

The displacements in *α*_2_ and *α*_3_ are

13.1 $${\omega }^{{\alpha }_{2}}={C}_{1}^{{\alpha }_{2}}r+\frac{{C}_{2}^{{\alpha }_{2}}}{{r}^{2}}+{C}^{{\alpha }_{2}}{\varepsilon }_{2}r$$

13.2 $${\omega }^{{\alpha }_{3}}={C}^{{\alpha }_{3}}({\varepsilon }_{3}-{\varepsilon }_{2})r+{C}^{{\alpha }_{2}}{\varepsilon }_{2}r$$

The boundary conditions used to determine the coefficients ${C}_{1}^{{\alpha }_{1}}$, ${C}_{2}^{{\alpha }_{1}}$, ${C}_{1}^{{\alpha }_{2}}$, ${C}_{2}^{{\alpha }_{2}}$ and ${C}^{{\alpha }_{3}}$ are

14.1 $${\lambda }_{1}{C}_{1}^{{\alpha }_{1}}-\frac{{\mu }_{1}}{{r}_{1}^{3}}{C}_{2}^{{\alpha }_{1}}={D}_{1}$$

14.2 $${\lambda }_{1}{C}_{1}^{{\alpha }_{1}}-\frac{{\mu }_{1}}{{r}_{2}^{3}}{C}_{2}^{{\alpha }_{1}}-{\lambda }_{2}{C}_{1}^{{\alpha }_{2}}+\frac{{\mu }_{2}}{{r}_{2}^{3}}{C}_{2}^{{\alpha }_{2}}=3{K}^{{\alpha }_{2}}({C}^{{\alpha }_{2}}-1){\varepsilon }_{2}$$

14.3 $${\lambda }_{2}{C}_{1}^{{\alpha }_{2}}-\frac{{\mu }_{2}}{{r}_{3}^{3}}{C}_{2}^{{\alpha }_{2}}=3{K}^{{\alpha }_{3}}({C}^{{\alpha }_{3}}-1)({\varepsilon }_{3}-{\varepsilon }_{2})$$

14.4 $${C}_{1}^{{\alpha }_{1}}{r}_{2}+\frac{{C}_{2}^{{\alpha }_{1}}}{{r}_{2}^{2}}-{C}_{1}^{{\alpha }_{2}}{r}_{2}-\frac{{C}_{2}^{{\alpha }_{2}}}{{r}_{2}^{2}}={C}^{{\alpha }_{2}}{\varepsilon }_{2}{r}_{2}$$

14.5 $${r}_{3}{C}_{1}^{{\alpha }_{2}}+\frac{{C}_{2}^{{\alpha }_{2}}}{{r}_{3}^{2}}={C}^{{\alpha }_{3}}({\varepsilon }_{3}-{\varepsilon }_{2}){r}_{3}$$

To simplify this expression, we use ${\lambda }_{1}=3{\lambda }^{{\alpha }_{1}}+2{\mu }^{{\alpha }_{1}}$, ${\mu }_{1}=4{\mu }^{{\alpha }_{1}}$, and so on. If we let *ε*_3_ = *ε*_2_, then Eqs (14.3) and (14.5) become

15.1 $${\lambda }_{2}{C}_{1}^{{\alpha }_{2}}-\frac{{\mu }_{2}}{{r}_{3}^{3}}{C}_{2}^{{\alpha }_{2}}=0$$

15.2 $${r}_{3}{C}_{1}^{{\alpha }_{2}}+\frac{{C}_{2}^{{\alpha }_{2}}}{{r}_{3}^{2}}=0$$

Generally, *λ*_2_ + *μ*_2_ ≠ 0 because *λ*_2_ and *μ*_2_ always are positive, so ${C}_{1}^{{\alpha }_{2}}={C}_{2}^{{\alpha }_{2}}=0$. Eqs (14.1)–(14.5) naturally turn into Eq. (9.1)–(9.3). It can prove the correction of Eqs (14.1)–(14.5).

Because ${C}^{{\alpha }_{2}}$ has been obtained from Eq. (10.3), ${C}_{1}^{{\alpha }_{1}}$, ${C}_{2}^{{\alpha }_{1}}$, ${C}_{1}^{{\alpha }_{2}}$, ${C}_{2}^{{\alpha }_{2}}$, and ${C}^{{\alpha }_{3}}$ are obtained from solving Eqs (14.1)–(14.5). Repeatedly, inserting a new core into the center of particle as before, and then setting up a new system of equations that are like Eqs (14.1)–(14.5).

In principle, we can use this method to obtain the strain field of the multilayered shell structure. However, the number of equations in these systems will increase with increasing numbers of layers in the shell structure of the particles, and solving these equations will become more laborious and complicated. Therefore, some simplifications will be made in this paper. First, we will assume that the elastic moduli of the particles are constants, i.e., independent of the position coordinates. Eshelby also used this hypothesis in his well-known papers^23^ because the hypothesis can greatly simplify the theoretical derivations. We shall see that our method also can be used for a condition of inhomogeneous elastic moduli provided that the elastic moduli are functions of the position coordinates. After using the hypothesis of homogeneous elastic moduli, a general system of equations can be written as follows:

16.1 $$\lambda {C}_{1}^{{\alpha }_{1}}-\frac{\mu }{{r}_{1}^{3}}{C}_{2}^{{\alpha }_{1}}={D}_{1}$$

162 $$\begin{array}{rrr}\lambda {C}_{1}^{{\alpha }_{1}}-\frac{\mu }{{r}_{2}^{3}}{C}_{2}^{{\alpha }_{1}}-\lambda {C}_{1}^{{\alpha }_{2}}+\frac{\mu }{{r}_{2}^{3}}{C}_{2}^{{\alpha }_{2}} & = & 3K({C}^{{\alpha }_{2}}-1)({\varepsilon }_{2}-{\varepsilon }_{1})\\  & \vdots  & \end{array}$$

16.n $$\lambda {C}_{1}^{{\alpha }_{n-1}}-\frac{\mu }{{r}_{n}^{3}}{C}_{2}^{{\alpha }_{n-1}}-\lambda {C}_{1}^{{\alpha }_{n}}+\frac{\mu }{{r}_{n}^{3}}{C}_{2}^{{\alpha }_{n}}=3K({C}^{{\alpha }_{n}}-1)({\varepsilon }_{n}-{\varepsilon }_{n-1})$$

16.s $$\lambda {C}_{1}^{{\alpha }_{s-1}}-\frac{\mu }{{r}_{s}^{3}}{C}_{2}^{{\alpha }_{s-1}}=3K({C}^{{\alpha }_{s}}-1)({\varepsilon }_{s}-{\varepsilon }_{s-1})$$

16s+1 $$\begin{array}{rrr}{C}_{1}^{{\alpha }_{1}}{r}_{2}+\frac{{C}_{2}^{{\alpha }_{1}}}{{r}_{2}^{2}}-{C}_{1}^{{\alpha }_{2}}{r}_{2}-\frac{{C}_{2}^{{\alpha }_{2}}}{{r}_{2}^{2}} & = & {C}^{{\alpha }_{2}}({\varepsilon }_{2}-{\varepsilon }_{1}){r}_{2}\\  & \vdots  & \end{array}$$

16s+n $$\begin{array}{rrr}{C}_{1}^{{\alpha }_{n-1}}{r}_{n}+\frac{{C}_{2}^{{\alpha }_{n-1}}}{{r}_{n}^{2}}-{C}_{1}^{{\alpha }_{n}}{r}_{n}-\frac{{C}_{2}^{{\alpha }_{n}}}{{r}_{n}^{2}} & = & {C}^{{\alpha }_{n}}({\varepsilon }_{n}-{\varepsilon }_{n-1}){r}_{n}\\  & \vdots  & \end{array}$$

162s-1 $${C}_{1}^{{\alpha }_{s-1}}{r}_{s}+\frac{{C}_{2}^{{\alpha }_{s-1}}}{{r}_{s}^{2}}={C}^{{\alpha }_{s}}({\varepsilon }_{s}-{\varepsilon }_{s-1}){r}_{s}$$

wherein subscript *s* is the innermost layer. In Eqs (16.2) and (16.2s-1), we bring in *ε*_1_ that is the stress-free strain of the outermost layer. Usually, we assume *ε*_1_ = 0 because we choose the outermost layer as the reference state. The 2*s*−1 unknown variables, ${C}_{1}^{{\alpha }_{1}}$, ${C}_{2}^{{\alpha }_{1}}$, …… ${C}_{1}^{{\alpha }_{s-1}}$, ${C}_{2}^{{\alpha }_{s-1}}$ and ${C}^{{\alpha }_{s}}$ must be determined from Eqs (16.1)–(16.2s-1); the other variables, ${C}^{{\alpha }_{n}}$ (*n* < *s*), have be obtained from the previous system of equations. We note that the system of equations Eqs (16.1)–(16.2s-1) can also extend to a particle embedded in an infinite matrix. At this case, Eq. (16.1) will be rewritten as

17.1 $$\lambda {C}_{1}^{{\alpha }_{1}}-\frac{\mu }{{r}_{1}^{3}}{C}_{2}^{{\alpha }_{1}}+\frac{\mu }{{r}_{1}^{3}}{C}^{M}={D}_{1}$$

and then we need to add a new equation

17.2 $${C}_{1}^{{\alpha }_{1}}{r}_{1}+\frac{{C}_{2}^{{\alpha }_{1}}}{{r}_{1}^{2}}-\frac{{C}^{M}}{{r}_{1}^{2}}=0$$

*C*^*M*^ is another variable that will be determined. In this paper, we will not discuss the case of the particle embedded in an infinite matrix.

We now deduce a general formula for these coefficients above when the discrete layers of the multilayer shell structure are transformed into a continuously changing layer structure. Firstly, we solve the coefficients of the first several layers of structures according to our previous discussion. For example, the coefficients, ${C}_{1}^{{\alpha }_{1}}$, ${C}_{2}^{{\alpha }_{1}}$ and ${C}^{{\alpha }_{2}}$, of two-layer structure are obtained which we have shown in Eqs (10.1)–(10.3); then substituting ${C}^{{\alpha }_{2}}$ into Eqs (14.2) and (14.4), and solving the system of Eqs (14.1)–(14.5), ${C}_{1}^{{\alpha }_{1}}$, ${C}_{2}^{{\alpha }_{1}}$, ${C}_{1}^{{\alpha }_{2}}$, ${C}_{2}^{{\alpha }_{2}}$ and ${C}^{{\alpha }_{3}}$ are obtained. Repeating this process will give the coefficients of the first several layers of structures. From these coefficients, we will find a general rule to determine the coefficients, ${C}_{1}^{{\alpha }_{n}}$, ${C}_{2}^{{\alpha }_{n}}$ and ${C}^{{\alpha }_{n}}$ of an arbitrary layer.

${C}_{1}^{{\alpha }_{n}}$, ${C}_{2}^{{\alpha }_{n}}$, and ${C}^{{\alpha }_{n}}$ are the functions of the radius of every layer, in addition their elastic constants. We can substitute *r*_1_ = *r*, *r*_2_ = *r* − Δ*r*, *r*_3_ = *r* − 2Δ*r* …… and *r*_*s*_ = *r* − (*s* − 1)Δ*r* into ${C}_{1}^{{\alpha }_{1}}$ and ${C}_{2}^{{\alpha }_{1}}$, *r*_1_ = *r* + Δ*r*, *r*_2_ = *r* and *r*_3_ = *r* − Δ*r* …… and *r*_*s*_ = *r* − (*s* − 2)Δ*r* into ${C}_{1}^{{\alpha }_{2}}$ and ${C}_{2}^{{\alpha }_{2}}$, ……, finally *r*_1_ = *r* + (*s* − 1)Δ*r*, *r*_2_ = *r* + (*s* − 2)Δ*r*, …… and *r*_*s*_ = *r* into ${C}^{{\alpha }_{s}}$. The coefficients ${C}_{1}^{{\alpha }_{n}}$ and ${C}_{2}^{{\alpha }_{n}}$ of the *nth* layer, and the ${C}^{{\alpha }_{s}}$ of the innermost layer are found in this manner. If we use the nomenclature Δ*ε*_*n*_ = *ε*_*n*_ − *ε*_*n* − 1_, and keep terms linear in Δ*r*, we have the following:

18.1 $${C}_{1}^{{\alpha }_{n}}=\frac{(\mu +3K){D}_{1}+3K\mu \sum _{i=2}^{s}{\rm{\Delta }}{\varepsilon }_{i}+9{K}^{2}\sum _{i=2}^{n}{\rm{\Delta }}{\varepsilon }_{i}-3K(\mu +3K)\sum _{i=2}^{n}{C}^{{\alpha }_{i}}{\rm{\Delta }}{\varepsilon }_{i}+{\theta }_{1}^{{\alpha }_{n}}{\rm{\Delta }}r}{(\lambda +\mu )(3K+{\eta }^{{\alpha }_{2}}{\rm{\Delta }}r)}$$

18.2 $${C}_{2}^{{\alpha }_{n}}={r}_{n}^{3}\frac{(\lambda -3K){D}_{1}+3K\lambda \sum _{i=2}^{s}{\rm{\Delta }}{\varepsilon }_{i}-9{K}^{2}\sum _{i=2}^{n}{\rm{\Delta }}{\varepsilon }_{i}-3K(\lambda -3K)\sum _{i=2}^{n}{C}^{{\alpha }_{i}}{\rm{\Delta }}{\varepsilon }_{i}+{\theta }_{2}^{{\alpha }_{n}}{\rm{\Delta }}r}{(\lambda +\mu )(3K+{\eta }^{{\alpha }_{2}}{\rm{\Delta }}r)}$$

18.3 $${C}^{{\alpha }_{s}}=\frac{{D}_{1}(1+\frac{3(s-1){\rm{\Delta }}r}{{r}_{s}})+3K\sum _{i=2}^{s}{\rm{\Delta }}{\varepsilon }_{i}-3K\sum _{i=2}^{s}{C}^{{\alpha }_{i}}{\rm{\Delta }}{\varepsilon }_{i}+{\theta }^{{\alpha }_{s}}{\rm{\Delta }}r}{3K{\rm{\Delta }}{\varepsilon }_{s}+\frac{3\lambda (s-1){\rm{\Delta }}r}{(\lambda +\mu ){r}_{s}}(\mu +3K){\rm{\Delta }}{\varepsilon }_{s}}$$

where,

19.1 $${\eta }^{{\alpha }_{n}}=\frac{3}{(\lambda +\mu )r}[(n-1)(\mu +3K)\lambda +(s-n)(\lambda -3K)\mu ]$$

192 $$\begin{array}{rl}{\theta }_{1}^{{\alpha }_{n}}\,= & \frac{3\mu (\mu +3K)(3K-\lambda )}{(\lambda +\mu )r}[\sum _{i=2}^{n-1}(n-i){C}^{{\alpha }_{i}}{\rm{\Delta }}{\varepsilon }_{i}+\sum _{i=n+1}^{s-1}(s-i){C}^{{\alpha }_{i}}{\rm{\Delta }}{\varepsilon }_{i}]\\  & +\frac{9K\mu (\mu +3K)}{(\lambda +\mu )r}[\sum _{i=2}^{n-1}(n-i){\rm{\Delta }}{\varepsilon }_{i}+\sum _{i=n+1}^{s-1}(i-n){\rm{\Delta }}{\varepsilon }_{i}]\\  & +\frac{3}{(\lambda +\mu )r}\{3K\mu (\lambda -3K)(s-n)\sum _{i=n+1}^{s-1}{\rm{\Delta }}{\varepsilon }_{i}\\  & -(\mu +3K)(n-1)[3K\lambda \sum _{i=2}^{n}{\rm{\Delta }}{\varepsilon }_{i}-\lambda (\mu +3K)\sum _{i=2}^{n}{C}^{{\alpha }_{i}}{\rm{\Delta }}{\varepsilon }_{i}]\}\\  & +\frac{3}{r}[(n-1)(\mu +3K){D}_{1}+3K\mu (s-n){\rm{\Delta }}{\varepsilon }_{s}]\end{array}$$

193 $$\begin{array}{rl}{\theta }_{2}^{{\alpha }_{n}}\,= & \frac{3\lambda (3K-\lambda )(3K+\mu )}{(\lambda +\mu )r}[(s-2n+1)\sum _{i=2}^{n}{C}^{{\alpha }_{i}}{\rm{\Delta }}{\varepsilon }_{i}+\sum _{i=n+1}^{s-1}(s-i){C}^{{\alpha }_{i}}{\rm{\Delta }}{\varepsilon }_{i}]\\  & -\frac{9K(3K-\lambda )}{(\lambda +\mu )r}[\lambda (s-2n+1)\sum _{i=2}^{s-1}{\rm{\Delta }}{\varepsilon }_{i}+\mu \sum _{i=2}^{n}(s-2n+i){\rm{\Delta }}{\varepsilon }_{i}]\\  & +\frac{3}{(\lambda +\mu )r}[\mu {(\lambda -3K)}^{2}\sum _{i=2}^{n}(s-2n+i){C}^{{\alpha }_{i}}{\rm{\Delta }}{\varepsilon }_{i}+3K\lambda (\mu +3K)\sum _{i=n+1}^{s-1}(i-2n+1){\rm{\Delta }}{\varepsilon }_{i}]\\  & -\frac{3(s-2n+1)}{r}[(3K-\lambda ){D}_{1}-3K\lambda {\varepsilon }_{s}]\end{array}$$

and,

19.4 $${\theta }^{{\alpha }_{s}}=\frac{3}{(\lambda +\mu ){r}_{s}}[\mu (\lambda -3K)\sum _{i=2}^{s-1}(s-i){C}^{{\alpha }_{i}}\Delta {\varepsilon }_{i}-\lambda (\mu +3K)(s-1)\sum _{i=2}^{s-1}{C}^{{\alpha }_{i}}\Delta {\varepsilon }_{i}]+\frac{9K}{(\lambda +\mu ){r}_{s}}[\lambda (s-1)\sum _{i=2}^{s}\Delta {\varepsilon }_{i}-\mu \sum _{i=2}^{s-1}(s-i)\Delta {\varepsilon }_{i}]$$

Eq. (18.3) is the equation describing ${C}^{{\alpha }_{i}}$. When Δ*r* → 0, and *s* → ∞, we have *s*Δ*r* = *r*_1_ − *r*_*s*_, and *i*Δ*r* = *r*_1_ − *r*_*i*_, where *r*_*s*_ is the radius of innermost layer, and *r*_*i*_ is the radius of the arbitrary *i*th layer. If the stress-free strain is a function of the radius, Δ*ε*_*n*_ = *ε*_*n*_ − *ε*_*n* − 1_ can be written as Δ*ε*_*n*_ = *ε*′(*r*_*n*_)Δ*r*. Then, *ε*′(*r*_*n*_) is the derivative of the stress-free strain. Eq. (18.3) may be written as an integral equation:

20 $${D}_{1}(3{r}_{1}-2{r}_{s})=3K{\int }_{{r}_{1}}^{{r}_{s}}[{r}_{s}+\frac{3\lambda ({r}_{1}-{r}_{s})+3\mu (r-{r}_{s})}{\lambda +\mu }][C(r)-1]\varepsilon \text{'}(r)dr+\frac{3\lambda \mu }{\lambda +\mu }{\int }_{{r}_{1}}^{{r}_{s}}({r}_{1}-r)C(r)\varepsilon \text{'}(r)dr$$

In Eq. (20) and the following one, we transform the discrete variables, ${C}^{{\alpha }_{i}}$, ${C}_{1}^{{\alpha }_{n}}$ and ${C}_{2}^{{\alpha }_{n}}$, into the continuous variables *C*, *C*_1_, and *C*_2_ respectively. Eq. (20) is a Volterra integral equation. We can transfer the Volterra integral equation into an ordinary differential equation^29^,

21.1 $$({A}_{1}x+{A}_{2}{r}_{1})\frac{d\phi }{dx}+{A}_{3}\phi +{A}_{4}\varepsilon ^{\prime} (x)+({A}_{5}x-{A}_{6})\varepsilon ^{\prime\prime} (x)=0$$

with boundary condition

21.2 $$\phi ({r}_{1})=\varepsilon ^{\prime} ({r}_{1})-\frac{2{D}_{1}}{3K{r}_{1}}$$

where *φ* = *Cε*′(*x*), and *A*_*i*_ (*i* =  1, 2, … 6) are constants related to elastic constants, and are written explicitly in appendix B. Eq. (21.1) can be solved with boundary condition if *ε*′(*x*) and *ε*′′;(*x*) are known. Substituting the solution of Eq. (21.1) into Eqs (18.1), (18.2), (19.2), and (19.3), *C*_1_ and *C*_2_ can be obtained.

221 $${C}_{1}=\frac{(\mu +3K)[3{r}_{1}-2r]{D}_{1}+3Kr\{\mu [\varepsilon ({r}_{s})-\varepsilon ({r}_{1})]+3K[\varepsilon (r)-\varepsilon ({r}_{1})]-(\mu +3K){\int }_{{r}_{1}}^{r}\varphi (x)dx\}+{{\rm{\Theta }}}_{1}(r)}{3Kr(\lambda +\mu )+3[\lambda (\mu +3K)({r}_{1}-r)+\mu (\lambda -3K)(r-{r}_{s})]}$$

222 $${C}_{2}={r}^{3}\frac{(\lambda -3K)[3{r}_{1}-2r]{D}_{1}+3Kr\{\lambda [\varepsilon ({r}_{s})-\varepsilon ({r}_{1})]-3K[\varepsilon (r)-\varepsilon ({r}_{1})]-(\lambda -3K){\int }_{{r}_{1}}^{r}\varphi (x)dx\}+{{\rm{\Theta }}}_{2}(r)}{3Kr(\lambda +\mu )+3[\lambda (\mu +3K)({r}_{1}-r)+\mu (\lambda -3K)(r-{r}_{s})]}$$

where the function Θ_1_(*r*) and Θ_2_(*r*) are defined as follows:

231 $$\begin{array}{ccl}{{\rm{\Theta }}}_{1}(r) & = & \frac{3\mu (\mu +3K)(3K-\lambda )}{\lambda +\mu }[{\int }_{{r}_{1}}^{r}(x-r)\phi (x)dx+{\int }_{r}^{{r}_{s}}(x-{r}_{s})\phi (x)dx]\\  &  & +\frac{9K\mu (\mu +3K)}{\lambda +\mu }[{\int }_{{r}_{1}}^{r}(x-r)\varepsilon \text{'}(x)dx+{\int }_{r}^{{r}_{s}}(r-x)\varepsilon \text{'}(x)dx]\\  &  & +\frac{9K\mu (\lambda -3K)}{\lambda +\mu }(r-{r}_{s})[\varepsilon ({r}_{s})-\varepsilon (r)]\\  &  & -\frac{3({r}_{1}-r)(\mu +3K)}{\lambda +\mu }\{[3K\lambda [\varepsilon (r)-\varepsilon ({r}_{1})]-\lambda (\mu +3K){\int }_{{r}_{1}}^{r}\phi dx]\}\end{array}$$

23.2 $${{\rm{\Theta }}}_{2}(r)=\frac{3\lambda (3K-\lambda )(3K+\mu )}{\lambda +\mu }[(2r-{r}_{1}-{r}_{s}){\int }_{{r}_{1}}^{r}\phi (x)dx+{\int }_{r}^{{r}_{s}}(x-{r}_{1})\phi (x)dx]-\frac{9K(3K-\lambda )}{\lambda +\mu }[\lambda (2r-{r}_{1}-{r}_{s})[\varepsilon ({r}_{s})-\varepsilon ({r}_{1})]+\mu {\int }_{{r}_{1}}^{r}(2r-{r}_{s}-x)\varepsilon ^{\prime} (x)dx]+\frac{3(\mu +3K)}{\lambda +\mu }[\mu (\lambda -3K){\int }_{{r}_{1}}^{r}(2r-{r}_{s}-x)\phi (x)dx+3K\lambda {\int }_{r}^{{r}_{s}}(2r-x-{r}_{1})\varepsilon ^{\prime} (x)dx]$$

When *C*, *C*_1_, and *C*_2_ are obtained, the restricted strains are

24.1 $${\varepsilon }_{rr}={C}_{1}-\frac{2{C}_{2}}{{r}^{3}}+{\int }_{{r}_{1}}^{r}C(x)\varepsilon ^{\prime} (x)dx$$

24.2 $${\varepsilon }_{\theta \theta }={\varepsilon }_{\phi \phi }={C}_{1}+\frac{{C}_{2}}{{r}^{3}}+{\int }_{{r}_{1}}^{r}C(x)\varepsilon ^{\prime} (x)dx$$

for *r*_1_ ≥ *r* > *r*_*s*_; and,

24.3 $${\varepsilon }_{rr}={\varepsilon }_{\theta \theta }={\varepsilon }_{\phi \phi }={\int }_{{r}_{1}}^{{r}_{s}}C(x)\varepsilon ^{\prime} (x)dx$$

for *r* < *r*_*s*_, and they are constants.

### Two simple examples

As applications of mathematical model in section Mathematical model, we will give two simple examples to check our derivation and explain our calculated process in this section.

#### Example 1:

*ε*(*r*) is a constant. In this case, the system becomes a two-layered shell-structure. The radius of innermost layer *r*_*s*_ = *r*_2_. *ε*(*r*) is written as

25 $$\varepsilon (r)=\{\begin{array}{ll}\varepsilon  & {r}_{s} < r < {r}_{1}\\ 0 & r < {r}_{s}\end{array}$$

and the derivative of *ε*(*r*) is

26 $$\varepsilon ^{\prime} (r)=\varepsilon \delta (r-{r}_{s})$$

where *δ*(*r* − *r*_*s*_) is Dirac delta function. Substituting Eq. (26) into Eqs (18)–(20), we have

27.1 $$C({r}_{s})=\frac{{D}_{1}(3{r}_{1}-2{r}_{s})+3K\varepsilon [{r}_{s}+\frac{3\lambda }{\lambda +\mu }({r}_{1}-{r}_{s})]}{3\varepsilon [K({r}_{s}+\frac{3\lambda }{\lambda +\mu }({r}_{1}-{r}_{s}))+\frac{\lambda \mu }{\lambda +\mu }({r}_{1}-{r}_{s})]}$$

27.2 $${C}_{1}({r}_{1})=\frac{{D}_{1}(\mu +3K)+3K\mu \varepsilon (1-3\frac{{r}_{1}-{r}_{s}}{{r}_{1}})}{3(\lambda +\mu )[K+\frac{{r}_{1}-{r}_{s}}{{r}_{1}}\frac{\mu (\lambda -3K)}{\lambda +\mu }]}$$

27.3 $${C}_{2}({r}_{1})={r}_{1}^{3}\frac{[{D}_{1}(\lambda -3K)+3K\lambda \varepsilon ](1-3\frac{{r}_{1}-{r}_{s}}{{r}_{1}})}{3(\lambda +\mu )[K+\frac{{r}_{1}-{r}_{s}}{{r}_{1}}\frac{\mu (\lambda -3K)}{\lambda +\mu }]}$$

Eqs (27.1)–(27.3) are the same as Eqs (10.1)~(10.3) if Δ*r* = *r*_1_ − *r*_*s*_ is a small quantity compared with *r*_1_, and only the linear terms of Δ*r* are kept. The results verify the correction of mathematical derivations in section Mathematical model.

#### Example 2:

$\varepsilon (r)$ is a linear function of radius $r$. In this case, we assume $\varepsilon (r)$ as

28 $$\varepsilon (r)=\{\begin{array}{c}0\\ -\frac{{\varepsilon }_{0}r}{{r}_{1}-{r}_{0}}+\frac{{\varepsilon }_{0}{r}_{1}}{{r}_{1}-{r}_{0}}\\ {\varepsilon }_{0}\end{array}\quad \begin{array}{c}r={r}_{1}\\ {r}_{0} < r < {r}_{1}\\ r < {r}_{1}\end{array}$$

where *ε*_0_ is the stress-free strain at *r* ≤ *r*_0_. The slope of the stress-free strain is a constant, i.e.$\varepsilon ^{\prime} =-\frac{{\varepsilon }_{0}}{{r}_{1}-{r}_{0}}$, and *ε*″(*r*) = 0. Eq. (21) be solved as

29 $$C(r)=-\frac{{A}_{4}}{{A}_{3}}+[\frac{{A}_{4}}{{A}_{3}}+\frac{\phi ({r}_{1})}{\varepsilon^ {\prime}}]{[\frac{{A}_{1}r+{A}_{2}{r}_{1}}{({A}_{1}+{A}_{2}){r}_{1}}]}^{-\frac{{A}_{3}}{{A}_{1}}}$$

Substituting *C* into Eq. (22.1) and (22.2), the other two parameters, *C*_1_, and *C*_2_, can be obtained. We do not write them here because their calculations are simple, but their expression is unduly long.

Before beginning the numerical calculation using our model, we will give some discussions about coefficient *C*. First, we consider a case which surface tension be ignored. In this case, *C* is independent of the stress-free strain. This result implies that the displacement and pressure in the innermost shell only are linear functions of the radius. Usually, the surface tension *f* of solids is about $\sim 1N/m$^30^. At the nanoscale, $-{D}_{1}=2f/{r}_{1}$ becomes comparable to *K*Δ*ε*^4^. The surface tension can occasions an extra compression strain that will neutralize a part of the tensile strain. For phase transformation with volume expansion, the nucleation barrier of phase transformation in nano-sized crystals increases due to the actions of interfacial tension^4^. At here, the restricted strain will be modified by the interfacial tension in nanoscale materials.

**Figure 2:**
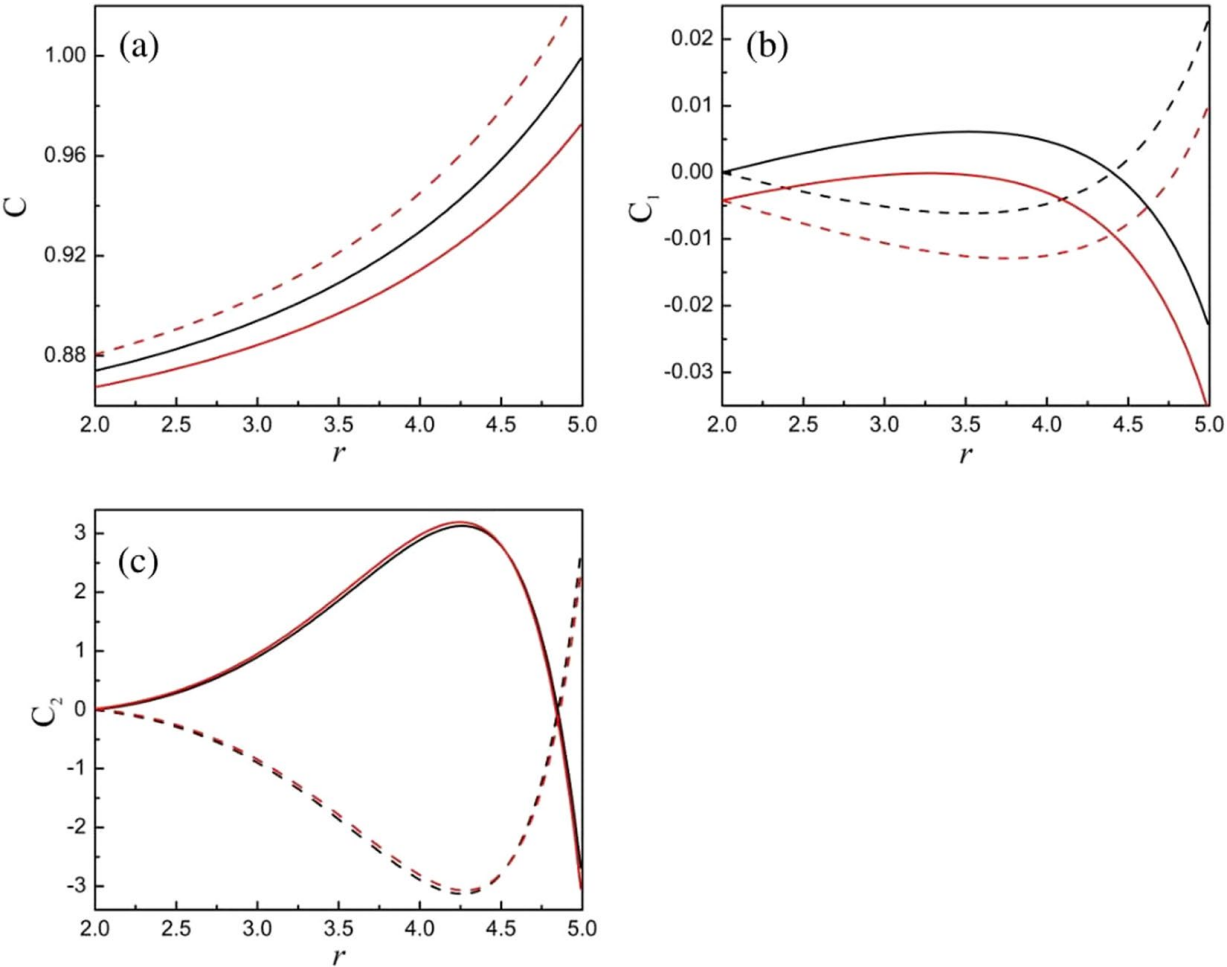
Calculated results for *C* (**a**), *C*_1_ (**b**), and *C*_2_ (**c**). The solid- and dashed-lines correspond respectively to *ε*_0_ = 0.05 and −0.05 in Eq. (28). The black lines represent *D*_1_ = 0, the red lines are for *D*_1_ = −0.01. Values of other required parameters are *K* = 1, *λ* = 1, *μ* = 1, *r*_1_ = 5, *r*_0_ = 2.

**Figure 3:**
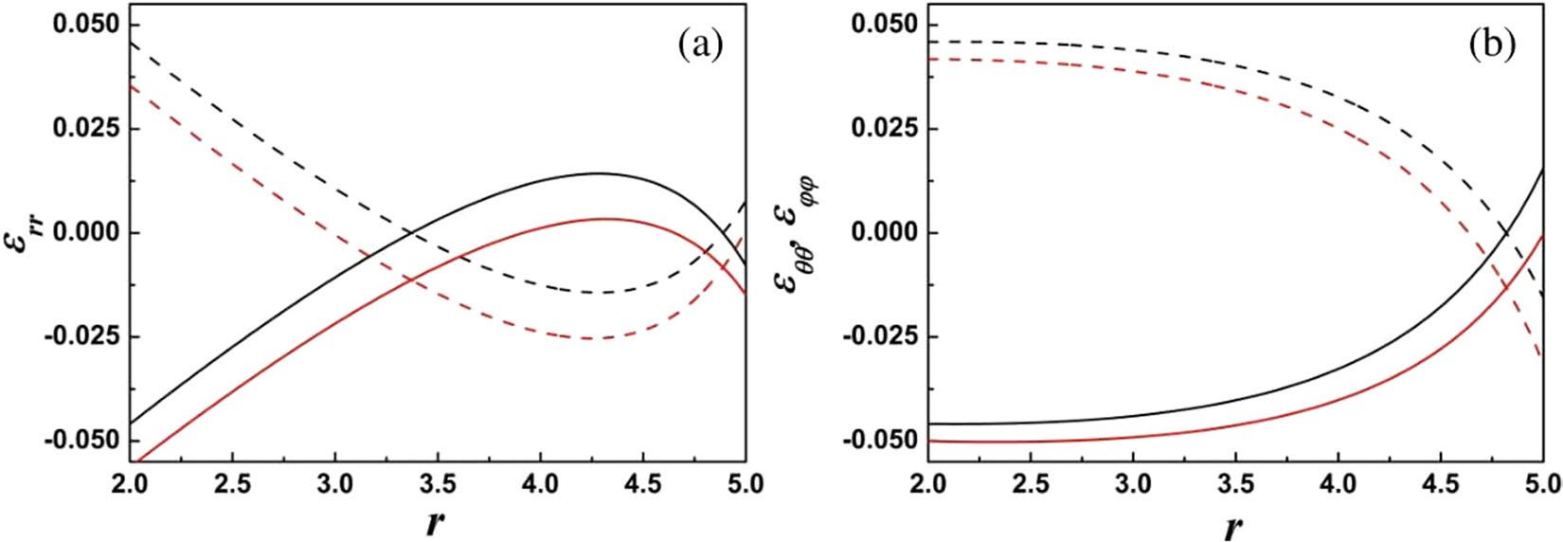
The restricted strains, using the results of Fig. (2). (**a**) Radial strain *ε*_*rr*_; (**b**) axial strain *ε*_*θθ*_ and *ε*_*φφ*_. The solid- and dashed-lines correspond *ε*_0_ = 0.05 and −0.05 respectively. The black lines are for *D*_1_ = 0, red lines for *D*_1_ = −0.01. The values of the other required parameters are same as in Fig. 2.

Figure 2 shows some calculated results of the variation of *C*, *C*_1_, and *C*_2_ with the stress-free strain in Eq. (28). The dashed- and solid-black-lines in Fig. 2(a) are merged into one line because *C* is independent on stress-free strain. From Fig. 2, we see these coefficients exhibit large changes near the particle’s surface. Figure 3 displays the variation of the restricted strains with radius using the *C*, *C*_1_, and *C*_2_ shown in Fig. 2. The difference of the change between *ε*_*rr*_ and *ε*_*θθ*_, *ε*_*φφ*_ will distort the lattice, such as from a cubic- to a tetragonal-shape. Our calculated results indicate that the strains are not monotonous function with radius. These results are consistence with the numerical calculations^21,22^. These calculated examples also tell us that the sign of the restricted strains may differ from the stress-free strains. For example, the restricted strain may be a compressive strain, although the stress-free strain is a tensile one in the area. Usually, some researchers estimate the change of chemical composition based on the located strain from observation of transmission electron microscopy. Our results indicate that this method will cause confusion.

Here, we should emphasize that Eq. (28) is only an artificial stress-free strain. An actual stress-free strain originated the non-uniform of chemical components or other variables of the system must lead to a minimum energy. For Li-ion battery, the Butler-Volmer equation determines the insertion of Li-ion at electrolyte/electrode interface and Cahn-Hilliard equation controls the diffusion of Li-ion in electrode^8,31–33^. In section Applications to Li-ion battery, we will calculate the evolution of strain-field during lithiation processing using phase field model and our strain-field theory, and discuss our calculated results.

### Inhomogeneous elastic moduli

Although we discussed only the case for homogeneous elastic moduli in section Mathematical model, our method can be extended easily to the inhomogeneous case. For non-uniform elastic moduli, we can write, such as …${\lambda }^{{\alpha }_{n-2}}={\lambda }^{{\alpha }_{n}}+2\frac{d{\lambda }^{{\alpha }_{n}}}{dr}{\rm{\Delta }}r$, ${\lambda }^{{\alpha }_{n-1}}={\lambda }^{{\alpha }_{n}}+\frac{d{\lambda }^{{\alpha }_{n}}}{dr}{\rm{\Delta }}r$, ${\lambda }^{{\alpha }_{n}}={\lambda }^{{\alpha }_{n}}$, ${\lambda }^{{\alpha }_{n+1}}={\lambda }^{{\alpha }_{n}}-\frac{d{\lambda }^{{\alpha }_{n}}}{dr}{\rm{\Delta }}r$, and ${\lambda }^{{\alpha }_{n+2}}={\lambda }^{{\alpha }_{n}}-2\frac{d{\lambda }^{{\alpha }_{n}}}{dr}{\rm{\Delta }}r$, in the expressions for ${C}^{{\alpha }_{n}}$, ${C}_{1}^{{\alpha }_{n}}$, and ${C}_{2}^{{\alpha }_{n}}$, and then, using the same process as described in section the mathematical model of non-uniformed strain, the sums or integrals of elastic moduli and derivatives of elastic moduli can be included in Eqs. (18.1–18.3). The rest of the process will be exactly the same as detailed in Section Mathematical model.

## Applications to Li-ion battery

The evolution of Li-ion concentration (*c*) inside the electrode can be determined from Cahn-Hilliard equation^7,8,33^:

30 $$\frac{\partial c}{\partial t}=\nabla Mc\nabla ({\rm{\Delta }}\mu )$$

and

31 $${\rm{\Delta }}\mu =\frac{\partial f}{\partial c}-\kappa {\nabla }^{2}c$$

where *M* is the mobility tensor (here taken to be isotropic and constant) and the internal chemical potential Δ*μ* derives from the homogeneous concentration dependent free energy *f* and the Cahn-Hilliard gradient energy coefficient *κ*^33^. The free energy includes the chemical free energy dependent on the chemical composition and the elastic energy arising from the lattice misfit-strain between Li-poor and Li-rich areas. In our simulation, we assume a maximum solubility of Li-ion is rescaled to one. For simplifying, two polynomial functions are used to describe the chemical free energy function in our calculation.

32.1 $${f}_{ch}(c)=a{(c-0.5)}^{2}$$

to describe the chemical free energy function of the case which only has one minimum point at *c* = 0.5. It means that the function describes a solid solution case without phase separation.

32.2 $${f}_{ch}(c)=a{(c-0.2)}^{2}{(c-0.8)}^{2}$$

to describe the case that has two minimum points at *c* = 0.2 and *c* = 0.8. It means that phase separation will occur at range 0.2–0.8 of Li-ion concentration. The coefficient *a* in Eq. (32) is a parameter to determine the value of chemical free energy. For the case of Eq. (32.2), the *a* determines the barrel between Li-poor *c* = 0.2 and Li-rich *c* = 0.8 phase.

The elastic energy is

33 $${f}_{el}(c)=\frac{1}{2}{C}_{ijkl}({\varepsilon }_{ij}-{\varepsilon }_{ij}^{0}(c))({\varepsilon }_{kl}-{\varepsilon }_{kl}^{0}(c))$$

where *C*_*ijkl*_ is the elastic constants which is assumed to be composition-independent here. ${\varepsilon }_{ij}^{0}(c)$ represents the composition-dependent stress-free strain of lithium compounds due to lattice expansion upon Li insertion. In this paper, we assume the unit cell volume has a linear Li concentration dependence, ${\varepsilon }_{ij}^{0}(c)={\rm{\Delta }}{\varepsilon }_{ij}^{0}c$, where $\Delta {\varepsilon }_{ij}^{0}$ is the linear misfit strain with maximum Li-ion concentration. For spherical symmetry and with the isotropic approximation, Eq. (33) becomes

34 $${f}_{el}(c)=(\frac{K}{2}-\frac{\mu }{3}){({\varepsilon }_{rr}+2{\varepsilon }_{\theta \theta }-3{\varepsilon }^{0}(c))}^{2}+\mu {({\varepsilon }_{rr}-{\varepsilon }^{0}(c))}^{2}+2\mu {({\varepsilon }_{\theta \theta }-{\varepsilon }^{0}(c))}^{2}$$

where *ε*_*rr*_ and *ε*_*θθ*_ will be calculated using theory of section: Mathematical model. Generally, Li-ion insertion on the electrode-electrolyte interface is determined by the Butler-Volmer equation^8,31,32^. Also for simplifying our calculation, we assume the rate of Li insertion is a constant; and once the concentration of Li-ion at the interface of electrode/electrolyte reach to one, it is held fixed.

Simulations are performed using the following non-dimensional forms of the length, energy and time units, a length scale *l*_0_ is angstrom, energy scale *E*_0_ is electron volt, and time scale ${t}_{0}=\frac{{l}_{0}^{5}}{M{E}_{0}}$. This yields a dimensionless radial position $\frac{r}{{l}_{0}}$, and dimensionless time $\frac{t}{{t}_{0}}$ . The evolution equations are solved numerically using an explicit, finite difference technique. The central difference formulae with fourth order error are used for the spatial derivative and backwards difference formula is used for the time derivative. Using parameters of materials in our simulations are listed in Table 1 and the elastic constants of Li_x_FePO_4_^34^ are used.

**Table 1:** List of Model Parameters to Using Our Calculation.

parameters	physical meaning	Values
K	Bulk modulus	93.9 GPa
G	Shear modulus	48.4 GPa
a	Coefficient of free energy function	1.6 × 10^12^ J/m^3^
κ	Concentration gradient coefficient	4.8 × 10^−9^ J/m
${\rm{\Delta }}{\varepsilon }_{ij}^{0}$	The maximum misfit strain	0.05

**Figure 4:**
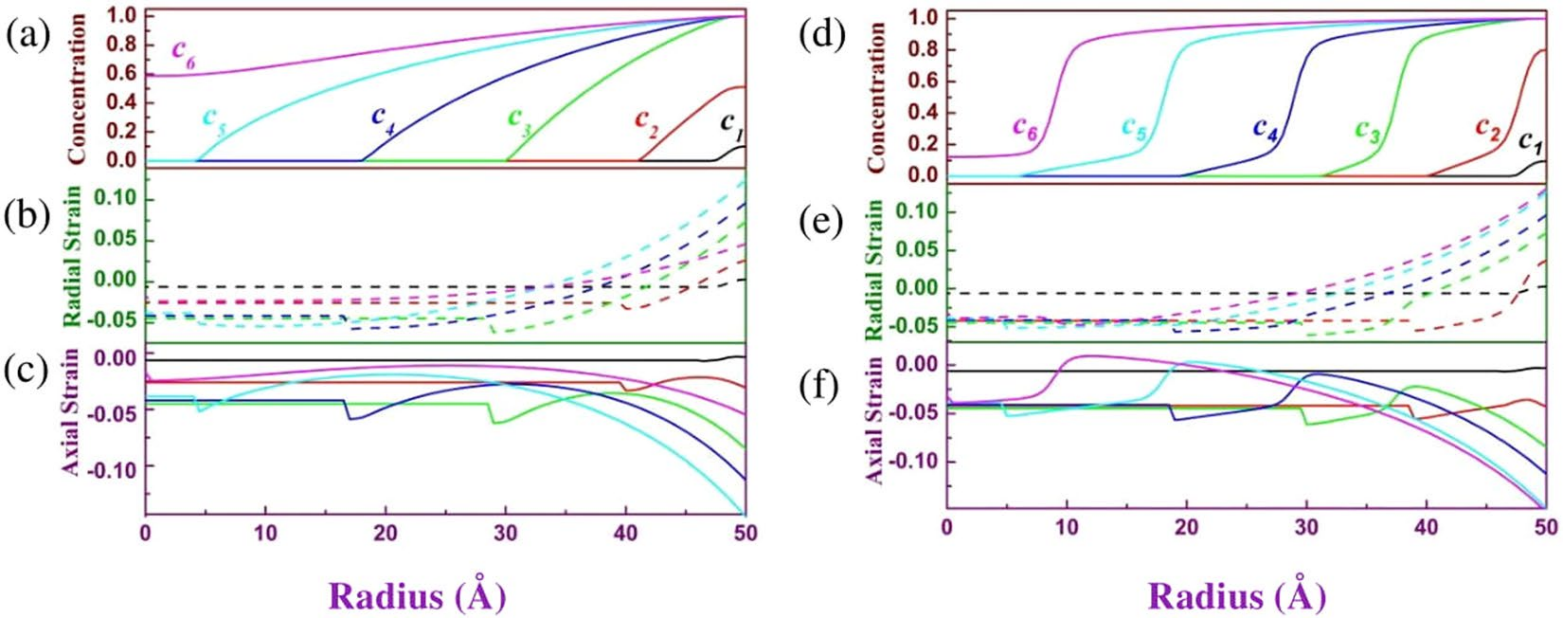
Radial distributions of the Li-ion concentration (first row), radial strain *ε*_*rr*_ (second row) and axial strain *ε*_*θθ*_ = *ε*_*φφ*_ (third row) using the free energy function (Eq. 32.1) (left column) and (Eq. 32.2) (right column) respectively. The *c1, c2,…*, and *c6* in (**a**) and (**d**) and corresponding colors symbolize six different average concentrations 0.0037, 0.074, 0.25, 0.42, 0.62 and 0.80 in whole sample.

Figure 4(a–f) show the two calculated results using different free energy functions: Eq. (32.1) (left column) and (32.2) (right column). The first row is concentration profile with various average concentrations in sample; the second and third rows are radial strain *ε*_*rr*_ and axial strain *ε*_*θθ*_ = *ε*_*φφ*_ respectively. We use different colors, black, red, green, blue, cyan and magenta, to respectively symbolize six different average concentrations, 0.0037, 0.074, 0.25, 0.42, 0.62 and 0.80, in Fig. 4. The curves of the radial strains in the middle row of Fig. 4 are plotted using dashed lines in order to distinguish the curves of concentration and axial strain. The continuous evolution of the concentration, radial strain and axial strain are shown in the video in Supplementary information. At the beginning stage of Li-ion insertion, the profile of Li-ion concentration and of strains is similar for the two free energy functions (Eq. 32.1 and 32.2). However, the evolutions of Li-ion concentration will become sensitively dependent on the free energy functions when Li-ion concentration is over 0.2 because of phase separation. For free energy function without phase separation (Eq. 32.1), the contour of concentration is gentle increase from Li-free to completely lithiation phase (see Fig. 4(a)). The width from Li-free to completely lithiation phase can cross the whole sample. However, for the free energy function with phase separation, a marked reaction front exists from Li-rich (more than 0.8 of Li-ion concentration) to Li-poor (less than 0.2 of Li-ion concentration) phase (see Fig. 4(d)), i.e., the phase separation occur. From our simulation, the strain curves of the two free energy functions are similar. The largest strain always occurs at surface of electrode and can exceed 15% (see Fig. 4(b,c) and (e,f)) although the maximum stress-free misfit strain is only 5%. The largest strain value at the surface is enough large to touch off crack of battery electrode of some materials.

**Figure 5:**
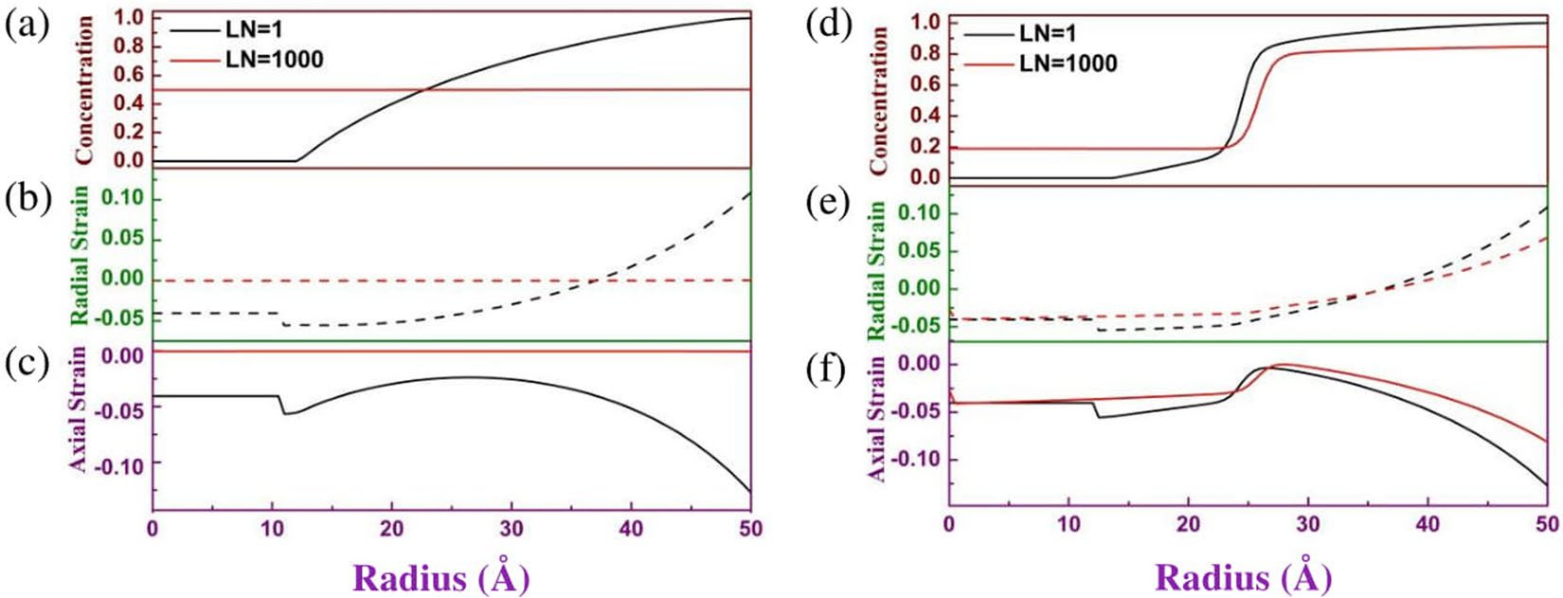
Radial distributions of the Li-ion concentration (first row), radial strain *ε*_*rr*_ (second row) and axial strain *ε*_*θθ*_ = *ε*_φφ_ (third row) with average concentration 0.5 in whole sample when the loop number (LN) is respectively 1 and 1000. Same as Fig. 4, the left column and right column respectively correspond to the free energy function (Eq. 32.1) and (Eq. 32.2).

The theory in the section Mathematical model tells us that the restricted strain inside sample is decrease with decreasing with the gradient of stress-free strain. For that reason, decreased the gradient of stress-free strain is advantageous to obtain small restricted strain and avoid the crack of electrode. Two factors can determine the gradient of stress-free strain: 1) the diffusion of Li-ion and 2) the rate of Li-ion insertion. In our program code, a variable, loop number (LN) for calculating Li-ion distribution after each step of Li-ion insertion in particle surface, can equivalently describe the diffusion coefficient of Li-ion or the rate of Li-ion insertion. Increasing LN means increasing the diffusion coefficient and decreasing the rate of Li-ion insertion. Experiments have proven that the scope of the diffusion coefficient of Li-ion at electrode may cover several orders of magnitude^35^. Figure 5 shows the profile of concentration and of strains with average concentration 0.5 in whole sample when LN is respectively 1 and 1000. LN from 1 to 1000 means the diffusion coefficient of Li-ion increases 1000 times or the rate of Li-ion insertion decreases 1000 times. Figure 5(a) indicates that the large LN can efficiently decrease the gradient of concentration in the sample without phase separation. The gradient of concentration almost approximate zero (red line of Fig. 5(a)) when LN is 1000, and then the corresponding restricted strain (see red line of Fig. 5(b) and (c)) is also near zero. However, for the sample with phase separation, a phase interface with great gradient of concentration (see Fig. 5(d)) always exists from Li-rich to Li-poor phase whatever the LN change. Therefore, the restricted strain cannot productively decrease with increasing LN. The results in Fig. 5(e) and (f) show that phase separation is disadvantage to prevent the crack of battery electrode although phase separation is good for obtaining a stable output voltage in Li-ion battery. However, comparing the red and black line in Fig. 5(e) and (f), the largest strain at the surface still decreases if the difference of concentration between Li-rich and Li-poor phase is small.

**Figure 6:**
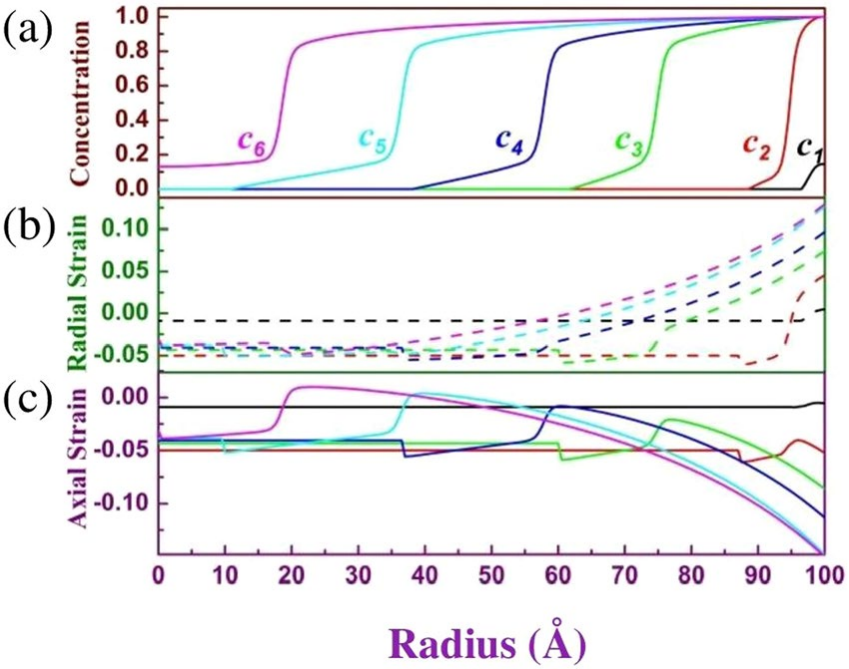
The evolution of concentration (**a**), radial (**b**) and axial strain (**c**) of particle radius 100 Å. The free energy function in the simulation is Eq. (32.2). Except particle size, other calculated parameters and average concentration *c1*, *c2* … and *c6* are same as Fig. 4.

In order to find the effect of particle size, the evolution of concentration and of strain in particle radius 100 Å are also calculated and shown in Fig. 6 using free energy function Eq. (32.2). Comparing the curves of the right column of Fig. 4 and Fig. 6, we surprisedly found that the evolution of strain with average concentration is almost unchanged with changing the particle size (Figures S1 and S2 that are similar to Fig. 6 in other particle radius 75 and 200 Å are supplied in Supplementary information). It means that the evolution of strain in sample is a function of ratio of Li-rich and Li-poor phase, not dependent on particle size. The result agrees with Christensen and Newman’s calculation^10,11^. However, Liu *et al*. and Ryu *et al*.’s experiments^15,16^ found there is a critical size below which the lithiation-induced strain can be accommodated without fracture in a Si electrode. Our results shown in Fig. 6 seem inconsistent with the experimental observations of Liu *et al*.^15^ and Ryu *et al*.^16^. The critical diameters of Liu *et al*.’s and Ryu *et al*.’s observations are respectively 150 nm and 300 nm. Obviously, the deviation of their observations about the critical diameters is big. According to our calculation, the largest strain at surface is mainly dependent upon the difference of concentration from surface to center of sample. The difference of concentration is controlled by the rate of Li-ion insertion, diffusion velocity of Li-ion and electrode size. If the Li-ions have diffused to the center of electrode before the largest strain at surface does not exceed the fracture strain, the electrode of this size will not crack because the difference of concentration between Li-rich and Li-poor regions will not increase. On the contrary, crack will produce. For their experiments, we can assume that the diffusion velocity of Li-ion in Si electrode is same in their experiment observations, but the rate of Li-ion insertion may be big different. The big difference of the rate of Li-ion insertion leads to the largest difference of concentration at different critical diameter. It is reason that Liu *et al*.^15^ and Ryu *et al*.’s^16^ observation is big different about the critical diameter. In addition, the shape of electrode probably brings about difference of the critical size since radial and axial diffusion is much different in nanowire^35^.

The fracture of electrode eliminates the useful capacity of battery associated with the growth of a passivating layer on the newly formed surface that leads to isolation of active materials from the conducting matrix^10,11^. The fracture of electrode can be observed in large volumetric swelling of up to 300% of the nonlithiated electrode materials, such as silicon and tin^15,16,36^, even has been found in commercial electrodes that undergo small volumetric expansion, for example LiCoO_2_, LiMn_2_O_4_ and LiFePO_4_^37–42^. However, Liu *et al*.^15^ have proven that silicon particles with diameter below 150 nm neither cracked nor fractured upon first lithiation although silicon undergoes ~280% volumetric expansion. On the other hand, the fracture can occur in the cubic phase of Li_x_Mn_2_O_4_ (x < 1)^40^ and LiFePO_4_^41^ in which the volume change is only 6.5% and 5%. It implies that large volume expansion due to Li-ion insertion is not a crucial reason to determine the crack of battery materials. Our theory and simulations confirm that the gradient of the stress-free strain dominates the maximum strain at surface of electrode; and the gradient of stress-free strain is only dependent on the gradient of the concentration, so making uniform distribution of Li-ion concentration is a key to prevent the crack of electrode. The large diffusion velocity, the slow rate of Li-ion insertion and the lithiation processing without phase separation in electrode is advantageous to prevent the crack of electrode.

## Conclusions

The non-uniform stress-free strain caused by change of chemical composition or phase transformation sensitively affects the microstructures of materials. It is crucial to understand the effect of non-uniform stress-free strain. We developed a new analytical method to calculate the restricted strain field in a spherically symmetric particle. Our analytical result shows that the gradient of stress-free strain in sample dominates the restricted strain field. The results of the numerical calculation using our theory indicate that the change of the restricted strain field is more complex than our expectation. Even when the stress-free strain is expansion, the restricted strain field can be compression in some areas and tensile in others. This means that we cannot simply use the measured strain to estimate the change of chemical composition.

Appling our theory to the lithiation process of Li-ion batteries, the evolution of strain with Li-ion insertion is calculated. The results indicate that the largest restricted strain always occurs at surface of electrode, and mainly depends upon the difference of Li-ion concentration between the surface and center of the electrode. If phase separation in the electrode does not occur during lithiation process, the larger diffusion velocity of Li-ion or the slower rate of Li-ion insertion can lead to a lesser difference in concentration from surface to center of the electrode; and then the maximum strain at surface of electrode is smaller. Otherwise, if phase separation occurs during lithiation, the difference of concentration both side of the phase interface is only dependent on the equilibrium concentration of two phases, no matter how fast the diffusion of Li-ion or how slow of Li-ion insertion. For large difference of equilibrium concentration of two phases, the large strain will produces at the surface of sample, suggesting the phase separation is disadvantageous to prevent the formation cracks in electrode. Our calculations also tell us that the fracture in an electrode is determined by not only particle size but also diffusion velocity of Li-ion or the rate of Li-ion insertion.

### Appendix A

The ${\varphi }_{k}^{(ij)}$ and ${\phi }_{i}^{(3)}$ in Eq. (10) are

A1 $${\phi }_{1}^{(3)}={\lambda }_{1}{\xi }_{1}^{(3)}$$

A2 $${\phi }_{2}^{(3)}={\mu }_{1}{\xi }_{2}^{(3)}$$

A3 $${\varphi }_{1}^{(31)}={\xi }_{1}^{(3)}$$

A4 $${\varphi }_{2}^{(31)}={\mu }_{1}{\varepsilon }_{2}$$

A5 $${\varphi }_{1}^{(32)}={\xi }_{2}^{(3)}$$

A6 $${\varphi }_{2}^{(32)}={\lambda }_{1}{\varepsilon }_{2}$$

A7 $${\varphi }_{1}^{(33)}={\mu }_{1}+{\lambda }_{1}$$

A8 $${\varphi }_{2}^{(33)}={\lambda }_{1}$$

A9 $${\varphi }_{3}^{(33)}={\mu }_{1}$$

where $${\xi }_{1}^{(3)}={\mu }_{1}-{D}_{2}$$, $${\xi }_{2}^{(3)}={\lambda }_{1}+{D}_{2}$$, $${\lambda }_{1}=3{\lambda }^{{\alpha }_{1}}+2{\mu }^{{\alpha }_{1}}$$, and $${\mu }_{1}=4{\mu }^{{\alpha }_{1}}$$.

### Appendix B

B1 $${A}_{1}=3K-\frac{3\lambda (3K+\mu )}{\lambda +\mu }$$

B2 $${A}_{2}=\frac{3\lambda (3K+\mu )}{\lambda +\mu }$$

B3 $${A}_{3}=-3K-\frac{3\lambda (3K+\mu )}{\lambda +\mu }$$

B4 $${A}_{4}=3K+\frac{9K\lambda }{\lambda +\mu }$$

B5 $${A}_{5}=-3K+\frac{9K\lambda }{\lambda +\mu }$$

B6 $${A}_{6}=-\frac{9K\lambda }{\lambda +\mu }$$

## Electronic supplementary material

Supplementary information

The evolution of concentration, radial and axial strain
