# Supplementary material for: Non-uniform Stress-free Strains in a Spherically Symmetrical Nano-sized Particle and Its Applications to Lithium-ion Batteries
Source: Sci Rep. 2018 Mar 21;8:4936. doi: 10.1038/s41598-018-23320-7 (PMC5862929; doi:10.1038/s41598-018-23320-7)

Supplementary Information:

Non-uniform Stress-free Strains in a Spherically Symmetrical Nano-sized Particle and Its Application to Lithium-ion Battery

Qingping Meng[1*], Lijun Wu[1], David O. Welch[1], Ming Tang[2], and Yimei Zhu[1*]

1. Brookhaven National Laboratory, Upton, New York, 11973
2. Department of Materials Science & Nanoengineering, Rice University

Figure S1 The evolution of concentration (a), radial (b) and axial strain (c) of particle radius 75Å. The free energy function in the simulation is Eq. 3.3(b). Except particle size, other calculated parameters and average concentration c_1, c_2 … and c_6 are same as Figure 4.

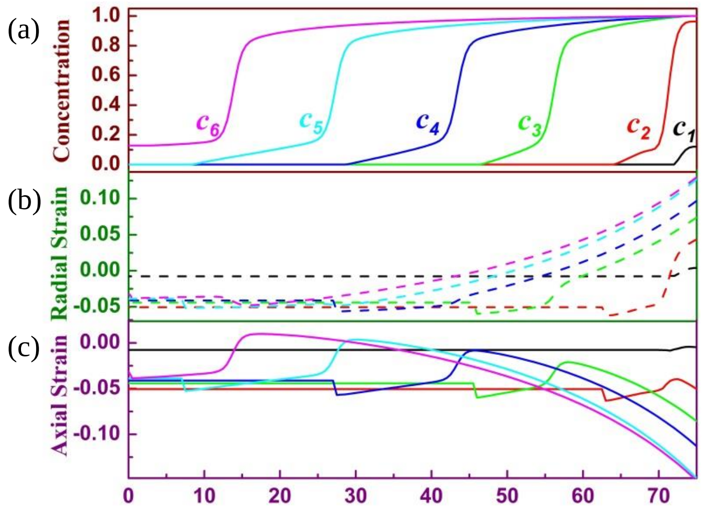

Figure S2 The evolution of concentration (a), radial (b) and axial strain (c) of particle radius 200Å. The free energy function in the simulation is Eq. 3.3(b). Except particle size, other calculated parameters and average concentration c_1, c_2 … and c_6 are same as Figure 4.

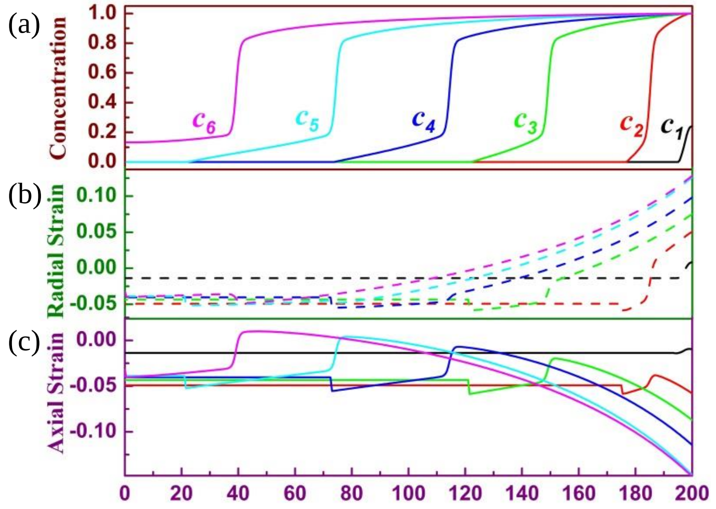

Supplement: Supplementary file 1 — Supplementary information [file 41598_2018_23320_MOESM1_ESM.pdf]
